# The Effects of Soy and its Components on Risk Factors and End Points of Cardiovascular Diseases

**DOI:** 10.3390/nu11112621

**Published:** 2019-11-01

**Authors:** Antonis Zampelas

**Affiliations:** Department of Food Science and Human Nutrition, Agricultural University of Athens, Iera Odos 75, 11855 Athens, Greece; azampelas@aua.gr

Cardiovascular diseases (CVD) remain the main cause of death in Westernized societies [1]. In Europe, CVD accounts for over 4 million deaths each year and it is responsible for the deaths of more women (2.2 million) than men (1.8 million), although CV deaths before the age of 65 years are more common in men (490,000 vs. 193,000) [2]. Prevention of CVD by dietary means is of paramount importance at both population and individual level. Recently, considerable attention has been given to the associated health benefits of soy, particularly regarding the reduction of CVD risk, mainly through its lowering effects on LDL-cholesterol levels. Much of the focus on soy had been directed toward the hypocholesterolemic properties of bioactive peptides in soy protein, in which it is believed that they exert effects on mechanisms involving the LDL receptor and bile acid regulation [3,4]. More recently, a meta-analysis of 46 controlled trials on which the Food and Drug Administration (FDA) could base its decision to revoke the heart health claim for soy protein was conducted [5]. Of the 46 trials identified by the FDA, 43 provided data for meta-analyses. Soy protein at a median dose of 25 g/d during a median follow-up of six weeks decreased LDL cholesterol by 4.76 mg/dL and decreased total cholesterol levels by 6.41 mg/dL compared with non-soy protein controls. At the same study, it was reported that soy protein significantly reduced LDL cholesterol by approximately 3–4% in adults. However, soy does not contain only protein, but it is also a significant source of phytochemicals such as isoflavones, phytosterols, and lecithins, as well as soluble fiber, saponins, and polysaccharides. Therefore, the effects of soy on CVD risk may not be solely attributed to its protein content and on its cholesterol lowering properties. 

A very interesting review article published in “Nutrients” tried to address these “pleiotropic” effects of soy [6]. In particular, Dam Ramdath and his co-workers reviewed the effects of soy on blood pressure, glucose levels, inflammatory markers, and obesity. They also addressed the non-protein effects of soy on blood lipids and they briefly reviewed its effects on satiety. The review was extensively referenced and it gave a variety of mechanisms through which soy exerts its beneficial actions. This thorough and in-depth review concluded that isoflavones and their metabolites may improve blood pressure, glycemic control, and inflammation. On the other hand, the review also suggested that the current evidence does not support hypotensive effects of other soy components such as protein, fiber, lecithins, and saponins. The review reported that the anti-adipogenic effects of isoflavones were mainly tested in animal models with some promising results, but soy protein did not appear to improve body composition greater than what is achieved with comparable levels of milk protein. Finally, it was also concluded that research looking at the effects of soy protein and other constituents on satiety is still limited. From this very balanced and extensive review, it was also apparent that well conducted randomized clinical trials (RCTs) examining the independent and in combination effects of these components on mainly end points of CVD are needed.

Following this extensive review, other studies and some interesting meta-analyses were conducted. One study systematically reviewed prospective cohort studies on the association between the consumption of soy products and mortality from all-causes, CVD, and cancer [7]. Seven studies were included for this meta-analysis. Three studies reported the risk of all-cause mortality and four studies assessed the risk of mortality from CVD and cancer. In total, 39,250 deaths were reported among 627,209 participants in a seven to eighteen-year follow-up. Unexpectedly, high consumption of soy products was not found to be significantly associated with a lower risk of mortality from all-causes and CVD. On the other hand, in a larger meta-analysis, the association between consumption of soy and risk of CVD (including stroke and coronary heart disease) was evaluated [8]. A total of 10 prospective cohort and seven case-control studies met the inclusion criteria. There were a total of 17,269 CVD events, including 6265 stroke events, 10,806 coronary heart disease events, and 198 other CVD events. A significant negative association was shown between soy intake and risk of CVD, stroke and coronary heart disease. However, no associations between soy isoflavones consumption and risk of CVD, stroke, and coronary heart disease were found. In another systematic review and dose-response meta-analysis of prospective studies results were presented on the associations between intakes of soy, soy isoflavones, and soy protein and risk of mortality from all causes, cancers, and cardiovascular diseases [9]. In total, 23 prospective studies with an overall sample size of 330,826 participants were included in this systematic review and the meta-analysis. Soy/soy products consumption was inversely associated with CVD. Participants in the highest category of dietary soy isoflavones intake had a 10% lower risk of all-cause mortality compared with those in the lowest category. However, intake of soy protein was not significantly associated with all-cause and cardiovascular diseases mortality. Lastly, an umbrella review of meta-analyses and systematic reviews of randomized trials and observational studies in humans aimed to assess the existing evidence of associations between consumption of soy and isoflavone and multiple health outcomes [10]. 114 meta-analyses and systematic reviews were identified with 43 unique outcomes. Soy and isoflavone consumption were found, among others, to exert beneficial associations for cardiovascular disease. The last three meta-analyses [7,8,9] and the umbrella review [10] indicate an inverse association between soy consumption and CVD. Additionally, there is a probable beneficial effect of isoflavones, but the beneficial effect of protein on end points of CVD cannot be fully confirmed yet.

In conclusion, recent evidence suggests an inverse association between of soy and end points of CVD. However, most of the studies are of prospective observational design and RCTs are necessary to confirm this association. The cause of this association is multifactorial. The published review [6] gives an in-depth knowledge of the mechanisms by which constituents of soy may affect risk factors of CVD.

## References

[1] World Health Organization (2011). Global Atlas on Cardiovascular Disease Prevention and Control: Policieas. Strategies and Intervention.

[2] Townsend N., Nichols M., Scarborough P., Rayner M. (2015). Cardiovascular disease in Europe—Epidemiological update 2015. Eur. Heart J..

[3] Torres N., Torre-Villalvazo I., Tovar A.R. (2006). Regulation of lipid metabolism by soy protein and its implication in diseases mediated by lipid disorders. J. Nutr. Biochem..

[4] Maki K.C., Butteiger D.N., Rains T.M. (2010). Effects of soy protein on lipoprotein lipids and fecal bile acid excretion in men and women with moderate hypercholesterolemia. J. Clin. Lipidol..

[5] Mejia S.B., Messina M., Li S.S., Viguiliouk E., Chiavaroli L., Khan T.A., Srichaikul K., Mirrahimi A., Sievenpiper J.L., Kris-Etherton P. (2019). A meta-analysis of 46 studies identified by the FDA demonstrates that soy protein decreases circulating LDL and total cholesterol concentrations in adults. J. Nutr..

[6] Dan Ramdath D., Padhi E.M.T., Sarfaraz S., Renwick S., Duncan A.M. (2017). Beyond the cholesterol-lowering effect of soy protein: A review of the effects of dietary soy and its constituents on risk factors for cardiovascular disease. Nutrients.

[7] Namazi N., Saneei P., Larijani B., Esmaillzadeh A. (2018). Soy product consumption and the risk of all-cause, cardiovascular and cancer mortality: A systematic review and meta-analysis of cohort studies. Food Funct..

[8] Yan Z., Zhang X., Li C., Jiao S., Dong W. (2017). Association between consumption of soy and risk of cardiovascular disease: A meta-analysis of observational studies. Eur. J. Prev. Cardiol..

[9] Nachvak S.M., Moradi S., Anjom-Shoae J., Rahmani J., Nasiri M., Maleki V., Sadeghi O. (2019). Soy, soy isoflavones, and protein intake in relation to mortality from all causes, cancers, and cardiovascular diseases: A systematic review and dose-response meta-analysis of prospective cohort studies. J. Acad. Nutr. Diet..

[10] Li N., Wu X., Zhuang W., Xia L., Chen Y., Zhao R., Yi M., Wan Q., Du L., Zhou Y. (2019). Soy and isoflavone consumption and multiple health Outcomes: Umbrella review of systematic reviews and meta-analyses of observational studies and randomized trials in humans. Mol. Nutr. Food Res..

